# Does Hemispheric Asymmetry Reduction in Older Adults in Motor Cortex Reflect Compensation?

**DOI:** 10.1523/JNEUROSCI.1111-21.2021

**Published:** 2021-11-10

**Authors:** Ethan Knights, Alexa M. Morcom, Richard N. Henson

**Affiliations:** ^1^Medical Research Council Cognition and Brain Sciences Unit, University of Cambridge, Cambridge CB2 7EF, England; ^2^Cambridge Centre for Ageing and Neuroscience, University of Cambridge, Cambridge, CB2 7EF, England; ^3^School of Psychology, University of Sussex, Brighton BN1 4GE, England; ^4^Department of Psychiatry, University of Cambridge CB2 3EB, England

**Keywords:** aging, compensation, HAROLD, life span, motor cortex, multivariate Bayes

## Abstract

Older adults tend to display greater brain activation in the nondominant hemisphere during even basic sensorimotor responses. It is debated whether this hemispheric asymmetry reduction in older adults (HAROLD) reflects a compensatory mechanism. Across two independent fMRI experiments involving adult life span human samples (*N* = 586 and *N* = 81, approximately half female) who performed right-hand finger responses, we distinguished between these hypotheses using behavioral and multivariate Bayes (MVB) decoding approaches. Standard univariate analyses replicated a HAROLD pattern in motor cortex, but in and out of scanner behavioral results both demonstrated evidence against a compensatory relationship in that reaction time measures of task performance in older adults did not relate to ipsilateral motor activity. Likewise, MVB showed that this increased ipsilateral activity in older adults did not carry additional information, and if anything, combining ipsilateral with contralateral activity patterns reduced action decoding in older adults (at least in experiment 1). These results contradict the hypothesis that HAROLD is compensatory and instead suggest that the age-related ipsilateral hyperactivation is nonspecific, consistent with alternative hypotheses about age-related reductions in neural efficiency/differentiation or interhemispheric inhibition.

**SIGNIFICANCE STATEMENT** A key goal in the cognitive neuroscience of aging is to provide a mechanistic explanation of how brain–behavior relationships change with age. One interpretation of the common finding that task-based hemispheric activity becomes more symmetrical in older adults is that this shift reflects a compensatory mechanism, with the nondominant hemisphere needing to help out with computations normally performed by the dominant hemisphere. Contrary to this view, our behavioral and brain data indicate that the additional activity in ipsilateral motor cortex in older adults is not reflective of better task performance nor better neural representations of finger actions.

## Introduction

Functional neuroimaging has established that increased age is linked to weaker task-based neural lateralization (Cabeza et al., 1997), with older adults showing increased activation of the nondominant hemisphere; a pattern summarized as hemispheric asymmetry reduction in older adults, (HAROLD; Cabeza, 2002). The explanation for this reduced lateralization is debated. A widely cited idea is that the recruitment of the nondominant hemisphere reflects compensatory mechanisms (Cabeza et al., 2018). An alternative hypothesis is that this increased activation is nonfunctional (Grady et al., 1994), perhaps reflecting inefficient or more dedifferentiated neural processing (Morcom and Johnson, 2015).

Motor responses, such as finger (Mattay et al., 2002; Rowe et al., 2006), wrist (Heuninckx et al., 2005), or grasping (Ward and Frackowiak, 2003; Ward et al., 2008) movements, are sufficient to evoke HAROLD patterns in motor areas. For example, mean activation within the right (ipsilateral) motor cortex increases with age when participants respond with their right hand (Tsvetanov et al., 2015). Brain–behavior relationships are commonly examined to adjudicate between the compensation and inefficiency hypotheses. If ipsilateral activity is compensatory, averaged activation will be positively related to behavioral performance. Nevertheless, univariate activation results are inconclusive: greater ipsilateral motor activation in older adults has been reported to show positive (Mattay et al., 2002; Heuninckx et al., 2008), negative (Langan, et al., 2010; Cassady et al., 2020), or no (Riecker et al., 2006) relationship with kinematics. Multivariate approaches offer an alternative way to test these competing hypotheses. If increased ipsilateral activity is compensatory (rather than nonfunctional), it should contain task-relevant information. Multivoxel pattern analysis (MVPA) has demonstrated that, in line with dedifferentiation, the distinctiveness of information represented within ipsilateral motor areas during finger tapping is reduced in older adults (Carp et al., 2011). However, a stronger assessment of whether ipsilateral motor activity is compensatory requires testing whether task-relevant information in ipsilateral cortex is complementary to that in contralateral cortex. The degree of complementarity could increase with age, even if the total amount of information in ipsilateral cortex decreases with age, as Carp et al. (2011) found (i.e., the greater information in young people in ipsilateral cortex could be redundant with that in contralateral cortex). This can be tested by combining voxels across hemispheres and testing whether decoding is improved relative to using voxels from the contralateral hemisphere alone (Morcom and Henson, 2018).

Morcom and Henson (2018) used multivariate Bayes (MVB), a model-based MVPA technique, to test whether one model (set of voxels) is more likely than another in predicting experimental conditions (Friston et al., 2008; Morcom and Friston, 2012). They tested a different aging-related hypothesis (posterior-to-anterior shift with age), which claims that increased anterior activity in older people is also compensatory (Davis et al., 2008). They found that when predicting memory, Bayesian model evidence in older adults was more often reduced rather than increased for a model with voxels from both anterior and posterior brain regions compared with a model with posterior voxels only. That is, results were more consistent with the hypothesis that age reduces the efficiency/differentiation of neural activity rather than compensation.

**Figure 1.**
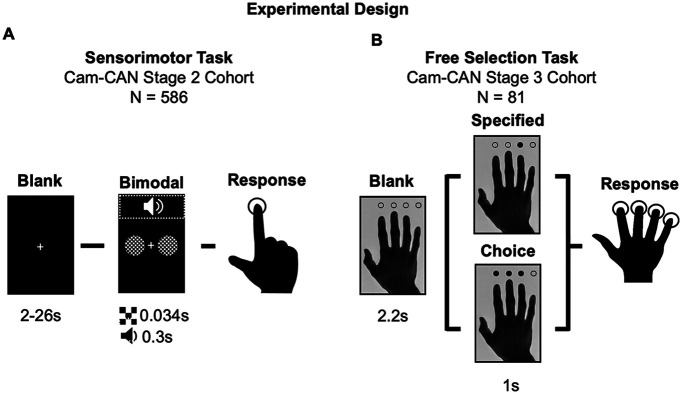
Experimental design. ***A***, Experiment 1. Sensorimotor task trials began with a blank fixation screen, followed by a bimodal (i.e., audio and visual) stimulus. Participants made finger-press responses if they sensed either or both types of stimulus. (There were also rare unimodal stimuli on ∼6% of trials, not shown here nor analyzed below, in which only an audio or visual stimulus was presented, whose purpose was just to ensure that both modalities needed to be attended.). ***B***, Experiment 2. Free selection task trials began with a picture of a hand with circles above the index, middle, ring, and little finger. Participants responded with a single finger press that matched one of the cued digits, where only one digit was cued in the specified condition, whereas in the choice condition, participants were free to choose one of the subset of three digits cued. Both experiments required right-hand responses only.

Here, we applied the same MVB logic to test HAROLD in the context of motor activity related to simple finger presses across two motor fMRI experiments in the Cambridge Center for Ageing and Neuroscience (Cam-CAN) population-derived adult life span sample (https://www.cam-can.org; Shafto et al., 2014). In experiment 1, participants (*N* = 586) pressed a button with their right index finger when they saw/heard a visual/auditory stimulus. In experiment 2, participants (*N* = 81) were cued to press the button under one of four fingers of their right hand (Fig. 1). First, we assessed whether greater mean ipsilateral sensorimotor cortex activation was associated with improved (i.e., shorter/less variable) reaction times for older adults during the scanner task and in separate tasks run outside the scanner. Second, we used MVB (and MVPA) to test whether the model evidence based on action decoding was boosted for older adults when models included ipsilateral voxels.

## Materials and Methods

### Experiment 1: sensorimotor task

#### Participants

A healthy population-derived adult life span human sample (*N* = 649; age approximately uniformly distributed from 18 to 87 years; females = 327, 50.4%) was collected as part of the Cam-CAN study (stage 2 cohort; Shafto et al., 2014). Participants were fluent English speakers in good physical and mental health based on the Cam-CAN cohort exclusion criteria, which excluded volunteers with a low Mini Mental State Examination score (≤ 24), serious current medical or psychiatric problems or poor hearing or vision, and based on standard MRI safety criteria. From this sample, we excluded participants who had missing behavioral measures from either in scanner (*N* = 4) or out of scanner (*N* = 44). We also excluded participants who responded to <90% of trials either in scanner (*N* = 10) or out of scanner (*N* = 5). Thus, the analyzed sample consisted of 586 participants (age range = 18–87 years; females = 292, 49.8%). The study was approved by the Cambridgeshire 2 (now East of England–Cambridge Central) Research Ethics Committee. Participants gave informed written consent.

#### Materials and procedure

The sensorimotor task involved 120 bimodal audio/visual trials, as well as eight unimodal trials (four visual and four auditory; Fig. 1*A*), which were included to discourage strategic responding to one modality only. Bimodal trials consisted of visual checkerboards presented on either side of a central fixation (34 ms duration) concurrently with a binaural auditory tone (300 ms duration). Unimodal trials consisted of either the isolated auditory or visual stimulus. The auditory tones were one of three equiprobable frequencies (300 Hz, 600 Hz, or 1200 Hz), which was not relevant to the task or current hypotheses. Participants were instructed to button press with the right-hand index finger when they heard or saw any stimuli. Each trial followed a fixation-only screen with a minimal stimulus onset asynchrony (SOA) of 2 s (resulting in SOAs ranging from 2 to 26 s) designed to optimize the estimation of the fMRI impulse response through a sequence of stimulation and null trials (Shafto et al., 2014).

#### Imaging data acquisition and preprocessing

The MRI data were collected using a Siemens Trio 3T MRI Scanner system with a 32-channel head coil. A T2*-weighted echo planar imaging sequence was used to collect 261 volumes, each containing 32 axial slices (acquired in descending order) with slice thickness of 3.7 mm and an interslice gap of 20% (for whole-brain coverage including cerebellum; repetition time = 1970 ms; echo time = 30 ms; flip angle = 78°; field of view = 192 mm × 192 mm; voxel size 3 × 3 × 4.44 mm). Higher resolution (1 mm × 1 mm × 1 mm) T1- and T2-weighted structural images were also acquired to aid registration across participants.

MR data preprocessing and univariate analysis were performed with SPM12 software (Wellcome Department of Imaging Neuroscience; https://www.fil.ion.ucl.ac.uk/spm), release 4537, implemented in the Automatic Analysis 4.2 pipeline (Cusack et al., 2015) described in Taylor et al. (2017). Specifically, structural images were rigid-body registered to a Montreal Neurological Institute (MNI) template brain, bias corrected, segmented, and warped to match a gray matter template created from the whole CamCAN Stage 2 sample using the DARTEL toolbox (Ashburner, 2007; Taylor et al., 2017). This template was subsequently affine transformed to standard MNI space. The functional images were spatially realigned, interpolated in time to correct for the different slice acquisition times, rigid-body coregistered to the structural image, transformed to MNI space using the warps and affine transforms from the structural image, and resliced to 3 mm × 3 mm × 3 mm voxels.

#### Univariate imaging analysis

To estimate activity for univariate voxelwise contrasts (i.e., to define ROIs), five conditions (i.e., three bimodal conditions, one per tone frequency and two catch conditions per audio or visual format) were distinguished within a general linear model (GLM) for each participant using SPM software. A regressor for each condition was created from δ functions aligned to the onset of a stimulus, which were convolved with the SPM canonical hemodynamic response function, plus the SPM temporal and dispersion derivatives, resulting in three regressors per condition. The null events were excluded from the model, and therefore all regression coefficients were defined relative to this baseline activity. Six additional regressors representing the three rigid body translations and rotations estimated in the realignment stage were included in each GLM to capture residual movement-related artifacts. Finally, the data were scaled to a grand mean of 100 over all voxels and scans within a session, and the model was fit to the data in each voxel. The autocorrelation of the error was estimated using an autoregressive(1)-plus-white-noise model, together with a set of cosines that functioned to high-pass filter the model and data to 1/128 Hz, that were estimated using restricted maximum likelihood. The estimated error autocorrelation was then used to prewhiten the model and data, and ordinary least squares was used to estimate the model parameters. Contrasts were used to average across the three tone frequencies in the bimodal trials (i.e., the rarer unimodal trials were not analyzed further). This model was used for ROI definition and MVB, whereas for regressions involving univariate data, we used a least-squares separate approach (Abdulrahman and Henson, 2016) before averaging over voxels.

#### Behavioral measures

Reaction time (RT) was the time from stimulus onset to button press onset. RTs were estimated during the fMRI sensorimotor task (i.e., in-scanner RT) and during an independent lab-based simple RT task (i.e., out-of-scanner RT) performed during Stage 1 of the Cam-CAN project. In the out-of-scanner task, participants were presented with the same picture stimulus as the free selection experiment (Fig. 1*B*; see below, Experiment 2: free selection, Materials and procedure) where, for each trial (*N* = 50), a blank circle above an index finger was filled black, cueing a button-press response to be performed as quickly as possible. On pressing the button (or after 3 s), the fill in the circle was cleared and followed by a pseudorandom intertrial interval (Shafto et al., 2014). Note that although the out-of-scanner task was speeded, the in-scanner task was unspeeded (so that older participants did not feel too challenged). For each participant, both the mean and SD (variability) of RTs across trials were computed.

### Experiment 2: free selection

#### Participants

Participants were a subset of the cohort in experiment 1 who also completed the Free Selection fMRI experiment during Stage 3 of Cam-CAN data collection (*N* = 87; ages approximately uniformly distributed from 19 to 85 years; females = 38, 43.7%). We excluded six participants whose out-of-scanner RT measures were not collected (all remaining participants responded to >90% of trials and were correct for >75% of trials). Therefore, the analyzed sample consisted of 81 participants (females = 35).

#### Materials and procedure

The free selection task was adapted from the three-choice free selection task of Zhang et al. (2012), which involves a visually paced right-hand button press task that is typically used to examine executive control and action decisions in aging. Across 240 trials, participants were presented with an image of a right hand and pressed a button with one finger in response to a cue (Fig. 1*B*). Individual trials involved either one of the circles (specified condition; *N* = 120, split equally between each of the four fingers) or three of the circles (choice condition; remaining 120 trials) being filled black. In both cases, participants were instructed to respond as quickly as possible with a single button press from a cued digit; thus for choice trials the responding finger could be freely selected. Cues were pseudorandomly ordered so that participants did not see four or more responses of the same condition in a row (Shafto et al., 2014). A short gap (either 4.2 s or 6.2 s) separated blocks of 20 trials.

#### Imaging data acquisition and preprocessing

Data acquisition and processing were the same as in experiment 1 (see above, Experiment 1: sensorimotor task, Imaging data acquisition and preprocessing), aside from an increased number of volumes being acquired (296) because of a longer session duration.

#### Univariate imaging analysis

The procedure described for experiment 1 was repeated here (see above, Experiment 1: sensorimotor task, Univariate imaging analysis), except that only the canonical HRF was used (because the blocked nature of trials prevents reliable estimation of the HRF derivatives (Henson, 2015)). For the present analyses, we combined onsets across the specified and choice conditions, leaving four predictors based on which finger was pressed (i.e., index, middle, ring, and little). These four conditions were averaged to estimate the mean response versus baseline.

#### Behavioral measures

The same variable definitions and computations were used as described for experiment 1 (see above, Experiment 1: sensorimotor task). Unlike experiment 1, the out-of-scanner RT variables were measured during a choice RT task with a design more comparable to the in-scanner free selection task. Specifically, the choice RT task had the same parameters as the simple RT task, but on each trial (*N* = 67) any one of the four circles above the fingers could be filled black, and the participant was instructed to press the corresponding finger as quickly as possible.

### General methods

#### Regions of interest

**Figure 2.**
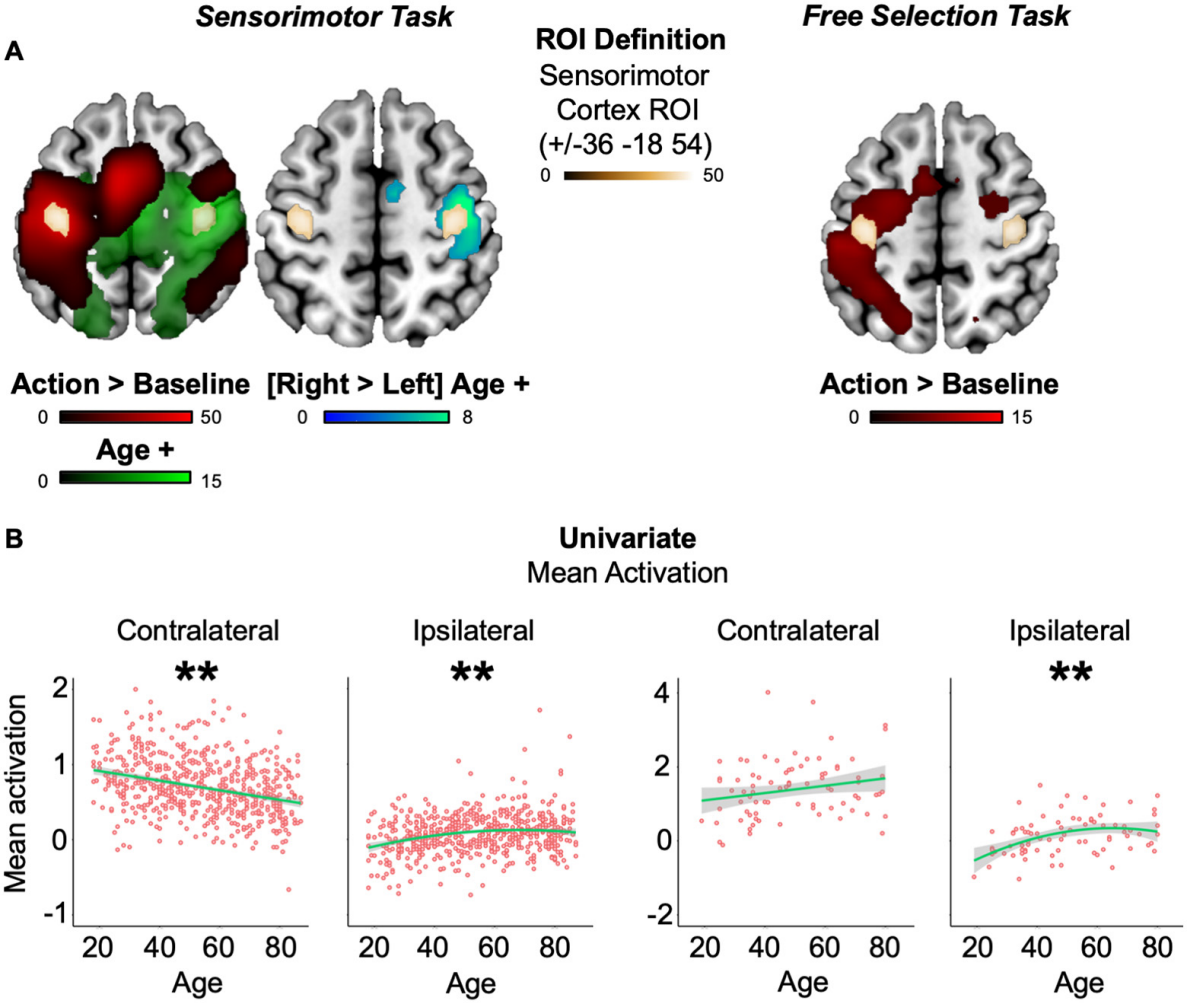
ROI definitions and responses. ***A***, ROI definitions. Univariate whole-brain voxelwise *t* tests are displayed on a standard template brain for all actions greater than baseline (red map) and for positive (linear) effect of age (green map). All colored voxels were corrected for multiple comparisons based on peak statistics using random field theory. The right map for experiment 1 shows results from a stricter lateralization analysis, where cyan-colored voxels show an age effect that was significantly more positive in the right than left hemisphere. Color depth indicates *t* statistic value. The actions greater than baseline contrast from experiment 1 was used to define the functional ROI in sensorimotor cortex (gold map), which was mirror flipped across hemispheres for unbiased analysis of age effects in both experiments (see above, Regions of interest). ***B***, Univariate ROI responses. Consistent with HAROLD, increased age predicted increased univariate activation of the ipsilateral ROI in both experiments, accompanied by the opposite pattern in the contralateral ROI. Green lines represent robust-fitted regression lines (with a second polynomial expansion in cases where a significant quadratic component was observed) and shaded areas show 95% confidence intervals. *p < 0.05, **p < 0.01.

A standard group univariate voxelwise approach was used to define a contralateral sensorimotor cortex region of interest (ROI), based on contrasting all bimodal trials versus baseline in experiment 1. Specifically, the 70 most significant voxels (based on *t* statistic rank) were selected according to the peak closest to the left hand knob landmark in the central sulcus (Yousry et al., 1997; Fig. 2*A*; Fig. 2*A*, Table 1, MNI coordinates). This contralateral ROI was mirror flipped (i.e., *x* coordinate reversed in sign) to create the ipsilateral sensorimotor cortex ROI (Fig. 2*A*; Table 1). Note that this ROI selection based on the average response versus baseline is averaged across age (i.e., not biased to show age effects). The same ROIs were applied to experiment 2 for consistency. Note that images were spatially smoothed (10 mm Gaussian kernel) for the purpose of ROI definition only. All ROI analyses used unsmoothed data. Additional results from a supplementary motor area (SMA) ROI are available on the Open Science Framework (see below, Data availability).

**Table 1.** Age effects on mean univariate and spread of multivariate action effects

Experiment	Measure/ROI		Age effect		Linear		Quadratic	
			*F* (*R*^2^)	*p*	*t* (β)	*p*	*t* (β)	*p*
Sensorimotor	Univariate mean	Contralateral	**31.7 (5.29)**	**<0.0001**	**−7.95 (−7.5)**	**<0.0001**	−0.3 (−0.29)	0.76
		Ipsilateral	**18.2 (2.89)**	**<0.0001**	**5.26 (1.22)**	**<0.0001**	**−3.11 (−0.72)**	**0.002**
	Multivariate spread	Contralateral	**4.82 (0.81)**	**0.008**	**2.31 (0.008)**	**0.021**	**−2.08 (−0.007)**	**0.038**
		Ipsilateral	2.97 (0.49)	0.052				
Free selection	Univariate mean	Contralateral	**4.49 (5.29)**	**0.014**	1.88 (1.34)	0.067	**−2.32 (−1.6)**	**0.025**
		Ipsilateral	**9.31 (10.2)**	**0.0002**	**3.66 (1.68)**	**0.0004**	**0.900 (−1)**	**0.032**
	Multivariate spread	Contralateral	2.83 (3.24)	0.065				
		Ipsilateral	2.02 (2.56)	0.14				

Effect sizes for the total age effect (linear and quadratic) and for the linear/quadratic effects separately are expressed as the proportion of explained variance (*R*^2^), as a percentage, and as standardized regression coefficients (β), respectively. Degrees of freedom in experiment 1: age effect *F*_(2,583)_ and *t*_(583)_; experiment 2: Age *F*_(2,78)_ and *t*_(78)_. Boldface indicates *p* < 0.05.

#### Multivariate Bayesian decoding

A series of MVB decoding models were fit to assess the information about actions represented in each ROI or combination of ROIs. Each MVB decoding model is based on the same design matrix of experimental variables used in the univariate GLM, but the mapping is reversed; many physiological data features (derived from fMRI activity in multiple voxels) are used to predict a psychological target variable (Friston et al., 2008). This target (outcome) variable is specified as a contrast. In both experiments, the outcome was whether an action had been performed (vs baseline), with all covariates apart from those involved in the target contrast (i.e., the null space of the target contrast) removed from both target and predictor variables.

Each MVB model was fit using a parametric empirical Bayes approach, in which empirical priors on the data features (voxelwise activity) are specified in terms of spatial patterns over voxel features and the variances of the pattern weights. As in earlier work, we used a sparse spatial prior in which patterns are individual voxels. Because these decoding models are normally ill posed (with more voxels relative to scans, or more precisely, relative to degrees of freedom in the time series), these spatial priors on the patterns of voxel weights regularize the solution. MVB also uses an overall sparsity (hyper) prior in pattern space that embodies the expectation that a few patterns make a substantial contribution to the decoding, and most make a small contribution.

The pattern weights specifying the mapping of data features to the target variable are optimized with a greedy search algorithm using a standard variational scheme, which iterates until the optimum set size is reached (Friston et al., 2007). This is done by maximizing the free energy, which provides an upper bound on the Bayesian log evidence (the marginal probability of the data given that model). The evidence for different models predicting the same psychological variable can then be compared by computing the difference in log evidences [equivalent to the log of the Bayes factor (BF); Friston et al., 2008; Chadwick et al., 2012; Morcom and Friston, 2012]. In this work, the main outcome measures were the log evidence for each model and the spread (SD) of weights across voxels in the ROI (Morcom and Henson, 2018).

To test whether ipsilateral activity was compensatory, we used a boost measure (Morcom and Henson, 2018) to assess the contribution of the ipsilateral ROI to performing actions. This used Bayesian model comparison within participants to assess whether a combined contralateral-ipsilateral (i.e., bilateral) model boosted prediction of actions relative to a contralateral-only model. The compensatory hypothesis, in which the ipsilateral hemisphere is engaged to a greater degree in older age and improves performance, predicts that a boost will be more often observed with increasing age. The dependent measure was the log model evidence coded categorically for each participant to indicate the outcome of the model comparison. The three possible outcomes were as follows: a boost to model evidence for bilateral relative to contralateral-only models (difference in log evidence > 3), ambiguous evidence for the two models (−3 < difference in log evidence < 3), or a reduction in prediction of action for bilateral relative to contralateral-only (difference in log evidence < −3). These values were chosen because a log difference of three corresponds to a Bayes factor >20, which is generally considered strong evidence (Lee and Wagenmakers, 2014).

For the across-participant analyses of this MVB boost, participants were only included if their data allowed reliable decoding by the bilateral model (Morcom and Henson, 2018). To determine this, we contrasted the evidence for the bilateral model with that from models in which the design matrix (and therefore the target variable) was randomly phase shuffled. One-tailed *t* tests were used to compare whether the mean difference between true and shuffled differences in log-evidence was >3 (Morcom and Henson, 2018; Fig. 4*A*), which ultimately left 582 and 54 participants from experiment 1 and 2, respectively (i.e., *N* = 4 and *N* = 27, excluded for log evidence < 3, respectively). For additional control analyses, we repeated the MVB boost analysis with models where voxel sizes were equated (see below, Results). For one of the control analyses that involved halving the number of voxels in the bilateral model, we repeated this preliminary phase-shuffling step because a different bilateral model was used, which led to excluding four additional participants in experiment 1 and prevented this particular control analysis for experiment 2 because there was not evidence that decoding was possible from this ROI (*p* = 0.12).

#### Multivoxel pattern analysis

Because MVB, as currently implemented by SPM, can only be applied to one-dimensional contrasts (e.g., between two conditions), we additionally used classical MVPA to decode which of the four fingers was pressed in experiment 2. Specifically, a multiclass support vector machine (SVM) was trained to decode the four fingers, using a one-versus-one coding design that was solved by binary learners. As classes were imbalanced, given that participants were able to choose which finger to respond with in the choice condition, we computed balanced decoding accuracies. For each ROI, a representative decoding accuracy was used to assess classifier performance based on averaging accuracies obtained from fourfold cross-validation, where the trials in each fold were defined randomly. Note that because the patterns were estimated from the same session (run), autocorrelation in the fMRI time series means that the patterns in the training and test sets are not independent, which could bias a MVPA classifier to produce above-chance classification (Mumford et al., 2014). However, we were only interested in the differences in classification performance across ROIs, which should not be compromised by this bias. Pattern classification was implemented with MATLAB fitcecoc functions using the provided default parameters. Beta estimates for each voxel were normalized (−1 to 1) across trials before input to the SVM (Smith and Muckli, 2010; Knights et al., 2021). The critical tests were whether decoding accuracy from ipsilateral hemisphere was predicted by age (Carp et al., 2011) and, like the MVB model comparison, whether there was an age-related boost to decoding accuracy when comparing bilateral and contralateral-only models. To keep the model comparison analysis similar between MVB and MVPA, we again excluded participants (*N* = 1) from the boost analysis if bilateral decoding accuracy was lower than chance (i.e., ≤25%) leaving 80 participants for the MVPA boost analysis.

#### Experimental design and statistical analysis

Age effects on continuous univariate, behavioral, and multivariate measures were tested using robust regression in R (version 3.6.1) with the rlm function (MASS package, version 7.3-51.4), to down weight extreme values (Venables and Ripley, 2002). These regression analyses used standardized linear and quadratic age predictors. Two-tailed robust *F* tests (Wald tests) were used to test the significance of regression coefficients. We first tested for general age effects (linear and/or quadratic), and if significant (α level of 0.05), we performed *post hoc* Wald tests on linear and quadratic age predictors separately. Analysis of the categorical outcomes for the between-region MVB model comparison (Fig. 4*B*) used ordinal regression. When all three categorical outcomes were observed, this was implemented with the polr function (MASS; as in Morcom and Henson, 2018; see Table 4), whereas glm (stats package, version 3.61) was used in binary cases (i.e., when reduction was not observed for any participant; Fig. 4*B*). For ordinal regression, the results are reported from a model containing only the linear age term, because of the categorical nature of the data, although the same pattern of findings was observed with the full quadratic model (see Table 4, with χ^2^ tests for general age effects). Standard effect sizes are reported for ordinal regression [odds ratios (OR)]. To maintain consistency between robust regression model statistics and effect sizes, we report individual standardized regression coefficients (β) and the proportion of unique weighted variance explained by age (*R*^2^), as a percentage, although the latter can inflate the coefficient of determination (compared with *R*^2^, e.g., from ordinary least-squares regression; Willett and Singer, 1988).

When important, null-hypothesis significance tests were supplemented with Bayes factors (Wagenmakers, 2007; Rouder et al., 2009). For continuous outcomes, we used the lmBF function (BayesFactor package, version 0.9.12–4.2) with default parameters (Rouder et al., 2012) to contrast models with and without the effect predicted by compensation accounts. For categorical outcomes (i.e., MVB model comparison), we used the brm function (brms package, version 2.10.0) with the Bernoulli family function to test for the absence of the hypothesis predicted by compensation (i.e., age effect > 0). A Student's *t* distribution prior was used, based on 7 degrees of freedom, a mean of 0, and a scale factor of 10 and 1 for the intercept and slope, respectively (Wagenmakers et al., 2010). The Bayes factors were interpreted according to criteria set out by Jeffreys, as cited in Jarosz and Wiley (2014), where a BF_01_ between 1 and 3, 3 and 10, and >10 indicates anecdotal, substantial, and strong evidence in favor of the null, respectively.

### Data Availability

Raw and minimally preprocessed MRI (i.e., from automatic analysis; Taylor et al., 2017) and behavioral data are available by request from Cam-CAN (https://camcan-archive.mrc-cbu.cam.ac.uk/dataaccess/). The univariate and multivariate ROI data and behavioral data can be downloaded from the Open Science Framework alongside analysis code (https://osf.io/seuz5/).

## Results

### HAROLD univariate effect

The univariate voxelwise contrast of key press versus baseline during the sensorimotor task (experiment 1), averaged across participants, showed strong contralateral activation throughout frontoparietal cortex (Fig. 2*A*, left, red voxels). The *x* coordinates of a contralateral motor cortex ROI that spanned suprathreshold voxels in the precentral gyrus were flipped to define an ipsilateral motor cortex ROI (Fig. 2*A*, gold voxels; Table 1; see above, Regions of interest). Although the ipsilateral motor cortex ROI was defined independently of age, it entirely overlapped voxelwise results from a positive *t*-contrast on the (linear) effect of age (Fig. 2*A*, left, green voxels). Furthermore, this ROI largely overlapped (87% of voxels) results from a stricter voxelwise analysis, which tested for voxels whose relationship with age was significantly stronger in one hemisphere than the other (i.e., by left-right flipping each participant's action > baseline image and subtracting this from their original image). The voxels showing a more positive age effect in the right than left hemisphere are shown in cyan in the right section of Figure 2*A* (No voxels showed a more positive age effect in the left hemisphere.). The only other motor region to show this lateralization was the right SMA (Fig. 2*A*), but further analyses revealed that the SMA showed negative effects of age in both hemispheres but just less negative in the right hemisphere. Because HAROLD is predicated on age-related hyperactivation, we did not analyze SMA further.

Consistent with the predictions of HAROLD, when averaging over voxels within the ipsilateral ROI, there was a significant effect of age on univariate activity with an increase in activation that flattened off in old age (Fig. 2*B*, left), which is in line with significant linear and quadratic components (Table 1). In fact, although the ROI was defined independently of age, it entirely overlapped voxelwise results from a positive *t*-contrast on the (linear) effect of age (Fig. 2*A*, left, green voxels). Further, this ipsilateral motor cortex ROI partially overlapped results from a stricter voxelwise analysis where we tested for a positive linear age effect for voxels that showed significantly greater activation in the right than left hemisphere (i.e., by subtracting a left-right flipped action > baseline map from the original map, per subject). In the contralateral motor cortex ROI, the significant age effect was in the opposite direction, with mean activity decreasing linearly as a function of age (Fig. 2*B*, left; Table 1).

When applying these ROIs defined in experiment 1 to experiment 2, we replicated this HAROLD effect, where greater age was associated with greater ipsilateral sensorimotor cortex activation, an age effect that again decelerated in later life (Fig. 2*B*, right; Table 1). Unlike experiment 1, no suprathreshold age effects were observed when repeating the voxelwise contrast (even if using a simpler contrast to test for a positive effect of age on the all actions > baseline contrast), possibly because of the lower statistical power than in experiment 1 (Fig. 2*A*, right). Again, the trend for the age effect in the contralateral ROI was in the opposite direction, although only the quadratic term was significant when tested independently (Table 1).

### Testing compensation: behavioral

**Figure 3.**
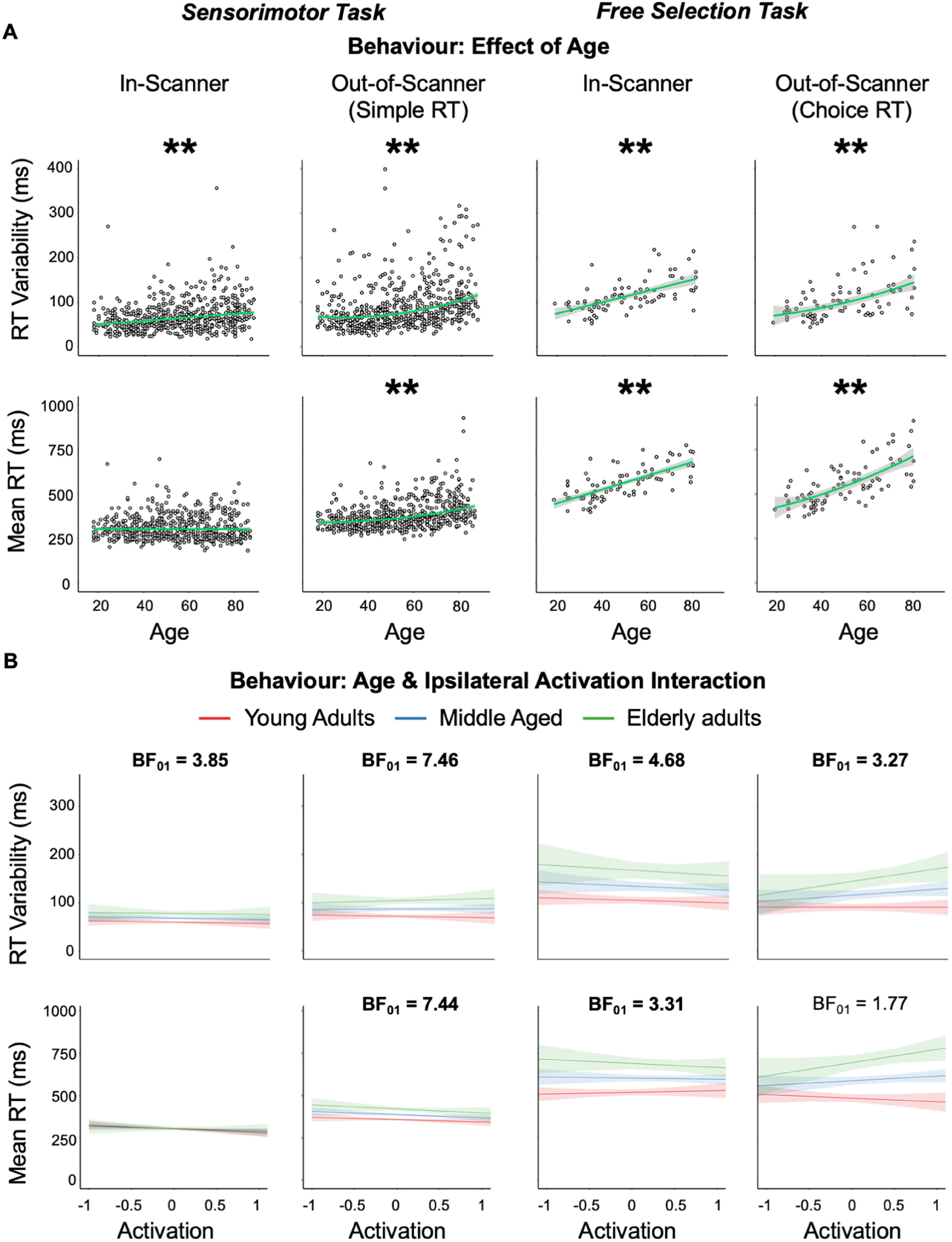
Behavioral results for RT variability and mean RT. ***A***, Effect of age. Increased age predicted worse performance (greater RT variability/mean) across experiments 1 (left) and 2 (right) whether measures were acquired in or out of scanner. For asterisk and regression line conventions, see Figure 2. ***B***, Interaction between age and ipsilateral univariate activation. No significant interactions between age and ipsilateral mean activation were observed across experiments, regardless of whether measures were acquired in or out of scanner. Bayes factors for the null (BF_01_) that had substantial evidence for this lack of interaction are in bold. Although the interaction was tested in a continuous fashion, tertile splits were used to define age groups (red, blue, green lines) for purposes of illustration.

If the increased univariate activity in ipsilateral sensorimotor cortex is compensatory, it might be expected to benefit task performance. We measured task performance using the variability (Table 2) and mean (Table 3) of RTs for both the in-scanner and out-of-scanner motor tasks. First, we tested whether there was a main effect of age on RT (Fig. 3*A*). For variability of simple RTs in experiment 1, significant effects were found for RTs recorded both in-scanner and out-of-scanner, where higher ages were linearly associated with increased variability, that is, worse performance (Table 2; Fig. 3*A*, left). These significant age effects were replicated in the choice RTs of experiment 2, both in-scanner and out-of-scanner, where, again, a linear positive change in RT variability was predicted by increased age (Table 2; Fig. 3*A*, right). For the out-of-scanner measure in experiment 1, the quadratic component was also significant, so the increase in RT variability actually accelerated in old age (Fig. 3*A*). For mean simple RTs, there was no significant effect of age for the in-scanner measure during experiment 1 (Table 3; Fig. 3*A*, left), most likely because this version of the task was not speeded. However, there was an age effect on the speeded out-of-scanner task like for RT variability with significant linear and quadratic components, indicating that worse performance accelerated in old age (Table 3; Fig. 3*A*, left). For experiment 2, the results for mean RT were similar to those reported for RT variability (i.e., there was a positive linear effect of age; Fig. 3*A*, right).

**Table 2.** Age and mean univariate effects from behavioral multiple regression with RT variability

Experiment	Measure/coefficient		Effect		Linear		Quadratic		BF01
			*F* (*R*^2^)	*p*	*t* (β)	*p*	*t* (β)	*p*	
Sensorimotor	In-scanner	Full model	**9.44 (1.69)**	**<0.0001**					
		Ipsilateral			−0.53 (−0.02)	0.596			
		Age	**22.9 (3.61)**	**<0.0001**	**6.66 (5.44)**	**<0.0001**	0.67 (0.53)	0.5	
		Ipsilateral* age	0.07 (0.01)	0.932					**3.85**
	Out-of-scanner	Full model	**20.7 (3.24)**	**<0.0001**					
		Ipsilateral			0.16 (0.005)	0.873			
		Age	**49.6 (7.84)**	**<0.0001**	**9.37 (6.31)**	**<0.0001**	**3.18 (2.1)**	**0.002**	
		Ipsilateral* age	0.49 (0.09)	0.612					**7.4**
Free selection	In-scanner	Full model	**11.3 (12.2)**	**<0.0001**					
		Ipsilateral			−0.75 (−0.07)	0.457			
		Age	**22.5 (23)**	**<0.0001**	**6.59 (5.61)**	**<0.0001**	1.02 (0.87)	0.319	
		Ipsilateral* age	0.36 (0.49)	0.698					**4.68**
	Out-of-scanner	Full model	**6.95 (7.84)**	**<0.0001**					
		Ipsilateral			0.65 (0.05)	0.512			
		Age	**10.4 (12.2)**	**0.0001**	**4.4 (3.68)**	**<0.0001**	0.47 (0.41)	0.641	
		Ipsilateral* age	1.15 (1.44)	0.321					**3.27**

Degrees of freedom for experiment 1: full model *F*_(5,580)_, age effects/interactions *F*_(2,580)_ and *t*_(580)_; experiment 2: full model *F*_(5,75)_, age effects/interactions *F*_(2,75)_ and *t*_(75)_. Boldface indicates *p* < 0.05 or Bayes factors > 3. See Table 1 for effect size conventions. Asterisks refer to interactions.

Having established age effects on task performance, the critical question was whether this age-related variance was related to ipsilateral motor activation, with compensation predicting that higher activation in older people would relate to better (i.e., faster and less variable) RTs. To assess this, we used multiple regression to test whether age, ipsilateral activation and their interaction predicted RT variability. If ipsilateral activity is compensatory and has an overall benefit to performance, then one would expect a significant interaction between age and ipsilateral activity, whereby the tendency for higher ipsilateral activation to be associated with reduced RT variability would increase with age. However, contrary to this prediction, no significant interaction between ipsilateral activity and age was observed when predicting RT variability (Table 2; Fig. 3*B*, top row) or mean RT (Table 3; Fig. 3*B*, bottom row) either in or out-of-scanner for experiment 1 or experiment 2. In fact, Bayes factors presented consistent evidence in favor of no interaction for all measures with a significant age effect in experiment 1 (Fig. 3*B*, left) and three of the four measures in experiment 2 (Fig. 3*B*, right; Tables 2, 3).

**Table 3:** Age and mean univariate effects from behavioral multiple regression with mean RT

Experiment	Measure/coefficient		Effect		Linear		Quadratic		BF_01_
			*F* (*R*^2^)	*p*	*t* (β)	*p*	*t* (β)	*p*	
Sensorimotor	In-scanner	Full model	2.03 (0.36)	0.074					
		Ipsilateral							
		Age							
		Ipsilateral* age							
	Out-of-scanner	Full model	**22.3 (3.61)**	**<0.0001**					
		Ipsilateral			−1.14 (−0.04)	0.258			
		Age	**54.38 (8.41)**	**<0.0001**	**10.03 (7.98)**	**<0.0001**	**2.43**	**0.015 (1.9)**	
		Ipsilateral* age	1.81 (0.36)	0.165					**7.44**
Free selection	In-scanner	Full model	**18 (18.5)**	**<0.0001**					
		Ipsilateral			0.4 (0.03)	0.693			
		Age	**33.4 (30.3)**	**<0.0001**	**7.64 (6.26)**	**<0.0001**	**2.07 (1.7)**	**0.044**	
		Ipsilateral* age	1.96 (2.56)	0.149					**3.31**
	Out-of-scanner	Full model	**16.2 (16.8)**	**<0.0001**					
		Ipsilateral			−0.57 (0.05)	0.565			
		Age	**23.4 (24)**	**<0.0001**	**6.68 (5.83)**	**<0.0001**	0.52 (0.48)	0.608	
		Ipsilateral* age	2.96 (3.61)	0.058					1.77

See Table 2 for degrees of freedom and conventions. Asterisks refer to interactions.

Finally, we tested the possibility that ipsilateral recruitment in later life partially compensates for reduced contralateral function. Although compensatory recruitment may have a net benefit to performance, compensation can also function like a walking stick, being engaged to a greater degree by people with a greater need for it (Bäckman and Dixon, 1992). In such cases, compensatory brain activity may correlate negatively with individual performance in older adults (i.e., only partially rather than fully compensating relative to younger people; Daselaar and Cabeza, 2005; de Chastelaine et al., 2011; Morcom and Johnson, 2015). We therefore used multiple regression to ask whether ipsilateral activation would relate positively to performance once effects of contralateral impairment were taken into account by including contralteral mean activity (i.e., degree of impairment) as a predictor. A partial compensation account of HAROLD would predict ipislateral activity to be associated with better performance only in people with low contralateral activity and not in people with maintained (high) contralateral activity (i.e., who did not need to compensate). This type of compensatory account therefore predicts an interaction between ipsilateral and contralateral activity in relation to RT performance (Compare the interaction between ipsilateral activity and age in the previous analyses.). To test this, we replaced the age predictor with contralateral ROI activity and ran this model on RT data that initially showed a significant effect of age (i.e., all RT measures except experiment 1 in-scanner mean RT; Fig. 3*A*). In neither experiment did we find a significant interaction between ipsilateral activity and contralateral activity (all *p* values ≥ 0.074), which would be suggested by partial compensation. This remained the case even if we added age as a third predictor. Indeed, there was substantial Bayesian evidence against this effect for all measures (with or without age) in experiment 1 (BF_01_ values ≥ 3.19) and in experiment 2, for the in-scanner RT variability measure (three-predictor model, BF_01_ = 4.04; all other BF_01_ values ≤ 2.98). Note that we observed main effects of contralateral activity for all measures (*p* values ≤ 0.048) excluding the experiment 2 out-of-scanner RT measures, which indicates that contralateral activity was generally a suitable proxy for age.

### Testing compensation: MVB

We further tested the compensation account of HAROLD using a multivariate approach. If the increasing ipsilateral activation with age reflected compensation, then multivoxel analyses should show that this increased activity carries additional information about actions, over and above that provided by the contralateral hemisphere. Note that this could happen even if the mean response across voxels did not relate to behavioral performance, as in the previous section (Morcom and Henson, 2018).

**Figure 4.**
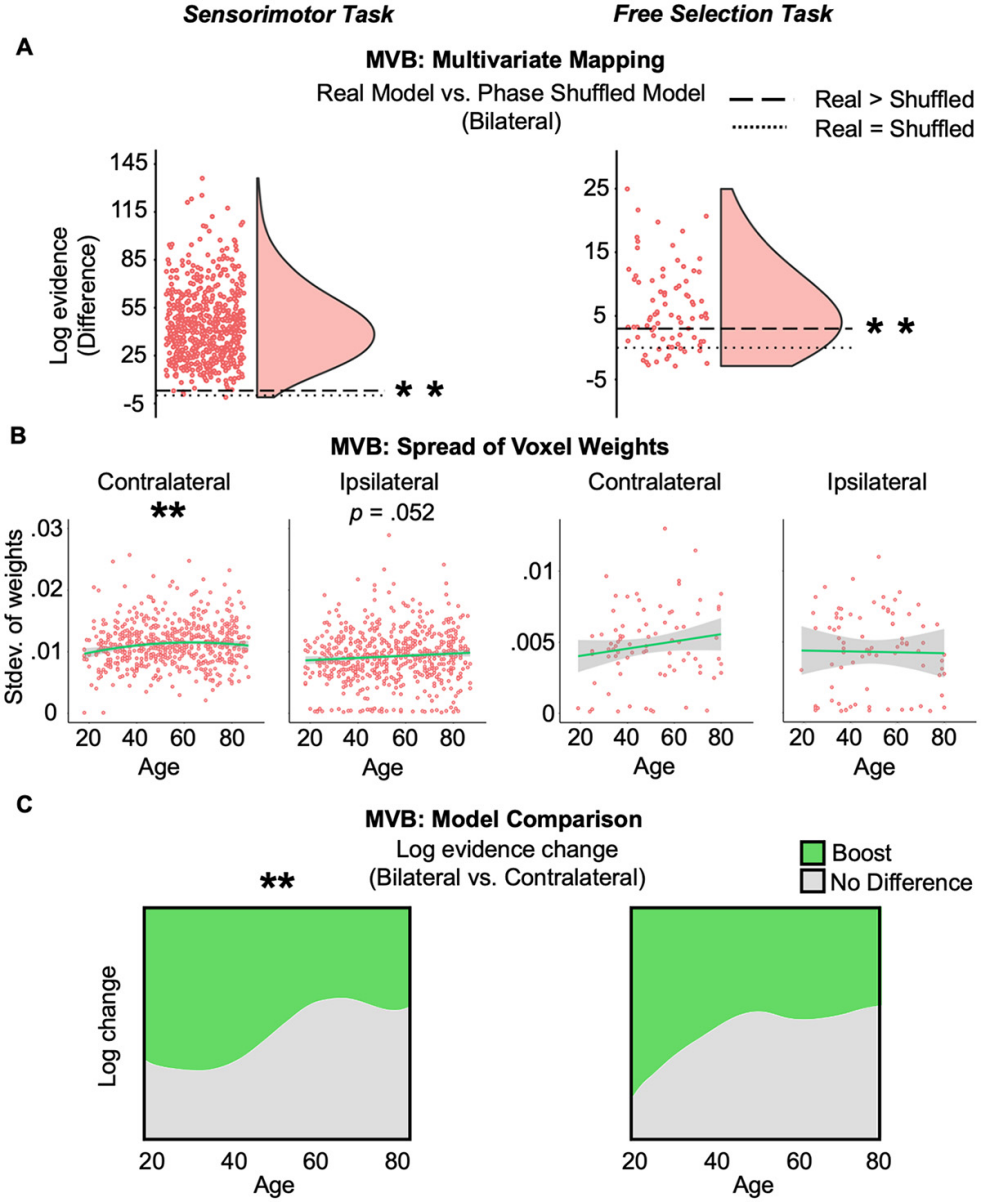
MVB results. ***A***, Multivariate mapping. For the target outcome being decoded (i.e., performing an action), the difference in log model evidence was significantly higher than 3 (dashed line) when using real (as opposed to phase shuffled) action onsets, indicating reliable decoding across both experiments (left and right; dotted line indicates a difference of 0). ***B***, Multivariate ROI responses. The spread of voxel weights showed an increase with age in the contralateral ROI in experiment 1, plus a similar trend in experiment 2 and for the ipsilateral ROI in experiment 1, but not in experiment 2. ***C***, Model comparison. Experiment 1 (left) results showed that contrary to a compensatory account, increased age actually led to a reduction in the likelihood of a boost when including ipsilateral voxels. For the free selection task (right), the effect of age was in the same direction but did not reach significance.

To test this, we first applied MVB to the combination of contralateral and ipsilateral motor ROIs (i.e., 138 voxels in total), to check that the classification of an action was above chance by comparing real versus phase-shuffled fMRI data. Results showed that the difference in log model evidence was >3 on average across participants in both experiment 1 (*t*_(585)_ = 44.27, *p* < 0.0001, *d* = 1.83) and experiment 2 (*t*_(80)_ = 4.57, *p* < 0.0001, *d* = 0.51). Figure 4*A* shows that decoding was possible for the majority of participants. There was also a significant linear effect of age on the probability that model evidence was (or was not) >3 for experiment 2, where successful decoding was more likely to occur for older ages (*z*_(80)_ = 3.11, *p* = 0.005, OR = 2.28). In experiment 1, this was not examined because of the rarity (*N* = 4) that the difference in model evidence was <3 (Fig. 4*A*).

Having shown that MVB decoding was possible, one measure of multivariate information is the spread (e.g., SD) of voxel classification weights (Morcom and Henson, 2018). This measure indexes the absolute magnitude of unique voxel contributions to the task. We therefore calculated spread for MVB models applied to each ROI separately. The results are shown in Figure 4*B*. In experiment 2, no significant effect of age was observed on the spread of either the contralateral or ipsilateral weights (Fig. 4*B*, right; Table 1). However, in experiment 1, there was a significant effect of age on spread for the contralateral ROI, in which the linear and quadratic components were significant, indicating that decodable information about a right finger press increased with age (in a decelerating fashion) in contralateral sensorimotor cortex (Fig. 4*B*, left; Table 1). The effect of age on weight spread was not significant for the ipsilateral hemisphere, although there was a trend in the same direction (Fig. 4*B*, left; Table 1). Thus, unlike in Morcom and Henson (2018), it might be that multivariate information about a right finger press increases with age in ipsilateral motor cortex, the region that is proposed to compensate. However, even if this age-related increase occurs for both ipsilateral and contralateral ROIs, it is possible that the same information is being represented in each hemisphere. That is, any age-related increase in information in the ipsilateral ROI could be redundant with that in the contralateral ROI rather than being unique (i.e., compensatory).

Therefore the crucial test was whether the information in the ipsilateral ROI improved action prediction compared with that in the contralateral ROI. Using MVB in experiment 1, the proportion of participants showing such an ipsilateral boost actually decreased rather than increased with age (linear, *z*_(580)_ = −2.86, *p* = 0.004; OR = 0.61; Fig. 4*C*). In other words, contrary to a compensatory account, the odds that model evidence was boosted by including ipsilateral with contralateral activity for older adults was 0.61 times that for younger adults. Indeed, the Bayes factor provided strong evidence in favor of accepting the null over the compensatory hypothesis (BF_01_ = 21.99). For experiment 2, no significant effect of age was found (*z*_(52)_ = −0.88, *p* = 0.38; Fig. 4*C*), although, in line with experiment 1, there was substantial Bayesian evidence against the compensatory hypothesis (BF_01_ = 4.87).

We performed a final check where we explicitly matched the number of voxels in the combined versus contralateral models. Regardless of whether we halved the number of voxels in the combined model (from 140 to 70), or doubled the number of voxels in the contralateral model (from 70 to 140), the significant linear negative effect of age in experiment 1 and nonsignificant effect in experiment 2 were replicated (after halving, experiment 1: *t*_(575)_ = −10.02, *p* < 0.0001; after doubling, experiment 1: *t*_(579)_ = −14.13, *p* < 0.0001; experiment 2: *p* = 0.29). All findings were of the same pattern across experiments when models contained both the linear and quadratic age terms (Table 4).

**Table 4.** Age effects (linear and quadratic) from ordinal regression MVB boost analyses

Experiment	Measure	Age effect	Linear			Quadratic		
		*p* (χ^2^)	*t* (OR)	*z* (OR)	*p*	*t* (OR)	*z* (OR)	*p*
Sensorimotor	Bilateral only, contralateral only	**0.021**		**−2.7 (<0.001)**	**0.007**		0.55 (11.7)	0.582
	Control: halve bilateral	**<0.0001**	**−7.21 (<0.001)**		**<0.0001**	−0.21 (0.67)		0.84
	Control: double contralateral	**<0.0001**	**−6.1 (<0.001)**		**<0.0001**	−1.43 (0.56)		0.151
Free Selection	Bilateral only, contralateral only	0.597						
	Control: halve bilateral							
	Control: double contralateral	0.49						

Effect sizes are presented as odds ratios for individual predictors.

### Testing compensation: MVPA

**Figure 5.**
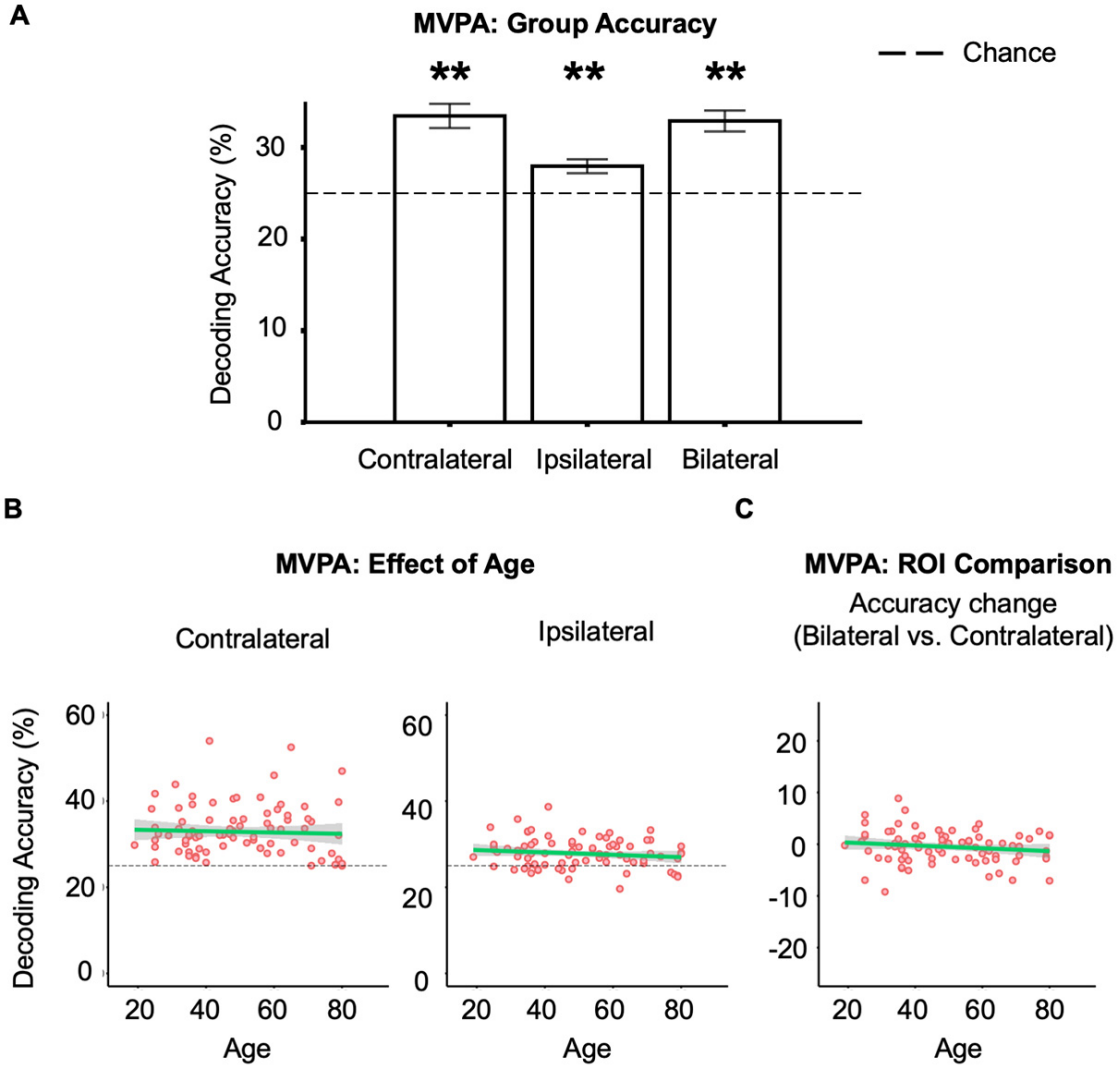
MVPA results for the Free Selection task. ***A***, Group accuracy. Mean decoding accuracy for each ROI across participants. Error bars represent ±2 SEM, **p < 0.01. Note that accuracy may be biased above chance because decoding was performed using data from the same fMRI run (see above, Multivoxel pattern analysis), but our interest here is the difference in accuracy between ROIs (as a function of age). ***B***, Effect of age. Decoding accuracy was not found to be significantly predicted by age. ***C***, ROI comparison. An age-related boost in decoding accuracy was not observed when comparing decoding accuracy from bilateral and contralateral-only ROIs.

Finally, we used standard MVPA to assess whether ipsilateral cortex activity contained additional information about which of the four fingers was being used to respond on a given trial (i.e., index, middle, ring vs little finger). This could only be run on experiment 2, where participants responded with different fingers. The mean classification results are shown in Figure 5*A*.

To assess the compensation hypothesis, we first examined whether decoding accuracy was predicted by age. No age effect was observed on decoding accuracy from either ROI (contralateral, *p* = 0.151; ipsilateral, *p* = 0.39; Fig. 5*B*). There was substantial evidence in favor of accepting this null effect of age for the contralateral ROI (BF_01_ = 4.31) but not for the ipsilateral ROI (BF_01_ = 1.6). Second, as in the key MVB analysis, we subtracted the multivariate information measure (in this case, decoding accuracy) of the bilateral from contralateral-only model to test whether adding ipsilateral voxels boosted the accuracy of between-finger prediction. Like for MVB, no age effect was found on the boost of decoding accuracy (*p* = 0.408), although for MVPA, Bayesian evidence was only weakly in favor of accepting the null (BF_01_ = 1.79). Also, like for MVB, control boost analyses again verified the same findings when the number of voxels between the contralateral and bilateral ROIs were matched, either by doubling the number of voxels in the contralateral ROI (*p* = 0.789) or halving those in the bilateral ROI (*p* = 0.176).

## Discussion

After replicating univariate HAROLD effects in motor cortex across two finger movement fMRI experiments in a large life-span sample, we tested if the additional ipsilateral activation in older adults reflected a compensatory mechanism. No behavioral or multivariate measures in either experiment showed age effects that would be predicted by a compensation account of HAROLD. In fact, Bayes factors demonstrated substantial evidence against compensatory interactions between age and ipsilateral mean activation for all behavioral analyses in experiment 1 as well as many in experiment 2. Likewise, the MVB boost analysis Bayes factors were strongly against positive age effects, where compensation accounts would predict an age-related boost for action decoding with additional ipsilateral voxels. For experiment 1, an age effect was even observed in the opposite direction; as age increased, adding the additionally activated voxels was found to be less likely to improve action decoding.

Previous tests of age-related compensatory accounts have been inconclusive (Ward, 2006). Some of this uncertainty might owe to differences in sample size, task and/or analysis. At least for finger presses, we believe that our sensorimotor results are more conclusive as they (1) come from relatively large and more population-representative samples, (2) simultaneously model age, behavior and (ispilateral and contralateral) activation, and (3) include a Multivariate Bayesian approach testing whether multivoxel information about actions in ipsilateral cortex is distinct (i.e., nonredundant) from that in contralateral cortex.

Another reason for the lack of agreement is that compensation may take more than one form (for review, see Scheller et al., 2014; Morcom and Johnson, 2015). Compensation may not always give rise to a positive relationship between compensatory activation and behavior. Instead, it might only be partially successful, analogous to a walking stick that helps older people walk faster than without it, but still not as fast as in the absence of age-related decline (Daselaar and Cabeza, 2005; de Chastelaine et al., 2011). Applied here, if performance declines with age because of reduced contralateral motor function, this may be only partially offset by compensatory ipsilateral activation, giving rise to net negative associations between ipsilateral activity and performance in older people. We therefore tested for partial compensation in additional behavioral analyses, where contralateral activity was a surrogate for the degree of age-related impairment. Still, there was evidence against the compensatory predicted interaction of contralateral and ipsilateral activity. Moreover, partial compensation is inconsistent with our MVB results, where multivariate information was more likely to be unchanged or reduced with the purported compensatory mechanism (i.e., ipsilateral activity) with increasing age.

Thus, our MVB analyses provide the strongest evidence against compensation (Fig. 4*C*). This is consistent with the only other multivariate experiment to our knowledge that examined this in the motor system, where MVPA demonstrated less distinctive ipsilateral motor cortex activity with age (Carp et al., 2011). However, our results strengthen that finding in a crucial way. Although age could reduce the information in ipsilateral motor cortex, it might also reduce information in contralateral motor cortex to a greater extent so that ipsilateral cortex still provides compensatory (nonredundant) information. This question of redundant information can only be tested by combining voxels across hemispheres, as enabled by MVB. Indeed, the voxel weight measure from MVB in experiment 1 hinted that older age might be associated with increased multivariate spread across hemispheres (Fig. 4*B*, left). Considered in isolation, this might support a compensatory role of ipsilateral motor cortex, contrary to Carp et al. (2011). However, MVB model comparison showed that adding these voxels did not lead to an age-related improvement in action decoding (i.e., this information was redundant to task performance, because it was already represented by the contralateral hemisphere). Indeed, this was consistent with standard MVPA decoding of which finger was pressed in experiment 2, which showed no additional multivariate information when combining the ipsilateral and contralateral motor cortex voxels.

If the HAROLD pattern does not reflect compensation, what does the age-related hyperactivation of ipsilateral sensorimotor cortex reflect? One possible explanation is neural inefficiency, where older adults simply require greater neural and/or hemodynamic activity for the same computation (for review, see Barulli and Stern, 2013). Alternatively, there is growing evidence of neural dedifferentiation, whereby the functional specificity of brain regions reduces with age, so additional areas (e.g., in the case of HAROLD, those that are ipsilateral) become involved in tasks that were not required when younger (for review, see Koen et al., 2020). Related to both ideas is the notion of task difficulty, illustrated by studies showing that younger adults activate similar additional areas to those of older adults but only for higher demands (Reuter-Lorenz and Cappell, 2008). Task difficulty indeed influences ipsilateral motor cortex activity differently with age (e.g., Seidler et al., 2004; Verstynen et al., 2005). The fact that we observed the inverse age effect during the boost analysis in experiment 1 (i.e., a simple detection task) but not experiment 2 (i.e., a more demanding, decision-making task) might be relevant (e.g., compensation occurs when the brain is confronted with difficult tasks), but this remains purely speculative because the difference could simply be attributed to power, given that the experiment 2 sample was an order of magnitude smaller.

Another noncompensatory account of HAROLD is motor disinhibition. Transcranial magnetic stimulation approaches have shown that movement-related motor cortex activity inhibits ipsilateral motor areas (Lee et al., 2003; Schambra et al., 2003; Sohn et al., 2003; Kobayashi et al., 2004; Vercauteren et al., 2008) and, crucially, that these mechanisms attenuate (Peinemann et al., 2001) or even reverse (Rowe et al., 2006; Talelli et al., 2008b) with age. In other words, increased ipsilateral activation could be the result of reduced interhemispheric/transcallosal inhibition (Ferbert et al., 1992; Lee et al., 2003; Plewnia et al., 2003; Naccarato et al., 2006; Talelli et al., 2008a; Langan et al., 2010; McGregor et al., 2011; Wang et al., 2016; Burianová et al., 2020). This is consistent with age-related disruption of corpus callosum integrity (Ota et al., 2006; Lenzi et al., 2007; Giorgio et al., 2010; Langan et al., 2010; Cox et al., 2015) and of functional connectivity between left and right motor cortices (Langan et al., 2010), as well as concentrations of glutamate (Kaiser et al., 2005) and GABA in these cortices (Cassady et al., 2019). Comparable inhibitory mechanisms have been proposed for memory (Logan et al., 2002; de Chastelaine et al., 2011), and for motor control, this provides plausible explanations of why older adults commit unintended mirror movements more often than younger adults (Koerte et al., 2010). This hypothesis could be examined by testing interhemispheric structural and functional connectivity in samples like Cam-CAN.

Finally, note that our results are based under constrained conditions (i.e., for motor cortex, during finger key presses, whether simple or choice) and might not apply to models that make comparable hypotheses about compensatory roles in frontal areas during cognitively taxing tasks (e.g., the Posterior-Anterior Shift in Aging (PASA) or Scaffolding Theory of Aging and Cognition (STAC) theories; Reuter-Lorenz and Park, 2014; but see Morcom and Henson, 2018). Better evidence for compensation within the HAROLD framework could come from more complex motor tasks that are known to evoke HAROLD effects in more widespread brain areas (e.g., grasping; Ward, 2006). Another limitation to consider is the degree to which age-related effects could be driven by increased noise in the fMRI data, for example because of greater (uncorrectable) head motion (Geerligs et al., 2017) or age-related changes in neurovascular coupling (D'Esposito et al., 2003). Although the simple explanation that some of our results are driven by noisier data in older adults might weaken the classical power to detect significant age effects, this would not explain the high Bayes factors we found for the null behavioral interactions (Fig. 4*B*). Likewise, if estimates were noisier in older adults, then successful decoding should have been less common for these participants; yet experiment 2 showed the opposite pattern, where the likelihood of successful decoding increased with age. It is possible that the age effects we found in ipsilateral sensorimotor cortex were purely vascular (e.g., because of weaker neurovascular coupling, a form of the inefficiency hypothesis discussed above), rather than neural. However, when adjusting task activations for resting-state fluctuation amplitudes, which are assumed to capture vascular reactivity, Tsvetanov et al. (2015) found that the increase of ipsilateral motor cortex with age in the same Cam-CAN data used for experiment 1 was one of few age-related effects to survive adjustment, suggesting it is not solely a vascular effect (Tak et al., 2021). Another limitation of the present study is that the sample was cross-sectional, which limits inferences to individual differences in birth year (and associated potential generational differences), rather than about the specific longitudinal changes that occur with age (Anstey et al., 2003). Future longitudinal studies could address this.

In conclusion, our behavioral and multivariate approaches both contradicted the hypothesis that HAROLD is compensatory. Instead, results suggested that at least in the case of ipsilateral motor cortex activity evoked by finger movements, this activation in older adults is nonspecific, perhaps reflecting neural inefficiency or motor disinhibition.
